# Molecular detection and phylogeny of *Hepatozoon* species in a wide spectrum of hosts from northwestern China

**DOI:** 10.3389/fvets.2026.1897152

**Published:** 2026-08-31

**Authors:** Xiaoshuang Han, Shujie Wang, Ziheng Liu, Xiaoping Yin, Weimin Xiahou, Quan Liu, Feng Tian, Yuanzhi Wang, Xiaobo Lu

**Affiliations:** 1 Infectious Disease and Liver Disease Center, The First Affiliated Hospital of Xinjiang Medical University, Urumqi, Xinjiang, China; 2 Key Laboratory of High Incidence Disease Research in Xinjiang (Xinjiang Medical University), Ministry of Education, Urumqi, Xinjiang, China; 3 Key Laboratory for Prevention and Control of Emerging Infectious Diseases and Public Health Security of the XPCC, School of Medicine, Shihezi University, Shihezi, Xinjiang, China; 4 NHC Key Laboratory of Prevention and Treatment of Central Asia High Incidence Diseases, Shihezi University, Shihezi, Xinjiang, China; 5 Xinjiang International Travel Healthcare Center (Urumqi Customs Port Outpatient Department), Urmqi, Xinjiang, China; 6 Shaoguan Customs Comprehensive Technology Service Center, Shaoguan, Guangdong, China; 7 Department of Infectious Diseases and Center for Infectious Diseases and Pathogen Biology, Key Laboratory of Organ Regeneration and Transplantation of the Ministry of Education, State Key Laboratory for Diagnosis and Treatment of Severe Zoonotic Infectious Diseases, Key Laboratory for Zoonosis Research of the Ministry of Education, The First Hospital of Jilin University, Changchun, Jilin, China; 8 Guangdong Key Laboratory of Animal Conservation and Resource Utilization, Institute of Zoology, Guangdong Academy of Sciences, Guangzhou, Guangdong, China

**Keywords:** diversity, *Hepatozoon*, molecular detection, northwestern China, wildlife

## Abstract

**Background:**

*Hepatozoon*, an apicomplexan, mainly infects amphibians, reptiles and mammals, accidentally causes anemia and icterus in human. Large-scale investigation remains lack. This study examined the occurrence of *Hepatozoon* spp. in a broad range of hosts in Xinjiang Uygur Autonomous Region (XUAR), northwestern China.

**Methods:**

During 2018–2025, 3,344 liver or blood specimens were collected from 3,037 mammals and 307 reptiles across XUAR (covering 1.66 million km^2^). Animal species were identified using morphology and mitochondrial gene amplification. *Hepatozoon* was identified by PCR targeting *18S rRNA* gene.

**Results:**

A total of 35 animal species were examined (25 mammals and 10 reptiles). The *Hepatozoon* infection rate was 2.5% (84/3344 specimens). All positives were exclusively in Rodentia (6.5%, 84/1301); no *Hepatozoon* DNA was found in the other six mammalian orders (Lagomorpha, Chiroptera, Artiodactyla, Perissodactyla, Carnivora, Primates) or Squamata reptiles. Combined with our previous report on *Hepatozoon canis* in the red fox (*Vulpes vulpes*) and *Hepatozoon felis* in the Eurasian lynx (*Lynx lynx*), the study detected five additional *Hepatozoon* species: *Hepatozoon chinensis*, *Hepatozoon ayorgbor*, *Hepatozoon ophisauri*, *Hepatozoon* cf. *ophisauri* and one unnamed *Hepatozoon* sp.

**Conclusion:**

This study provides the first reports of *Hepatozoon* infection in several rodent species: *Hepatozoon chinensis* in the root vole (*Microtus oeconomus*), *H*. *ayorgbor* in the five-toed jerboa (*Allactaga sibirica*), *H*. *ophisauri* in the mole vole (*Ellobius talpinus*) and five-toed jerboa, *H*. cf. *ophisauri* in the great gerbil (*Rhombomys opimus*) and Libyan jird (*Meriones libycus*), and *Hepatozoon* sp. in the long-tailed ground squirrel (*Spermophilus undulatus*). *Hepatozoon* surveillance on more wildlife should be strengthened.

## 1 Introduction

*Hepatozoon* (Eucoccidiorida: Adeleorina: Hepatozoidae), belonging to phylum Apicomplexa, is characterized by the invasion of host erythrocytes and leukocytes, and can infect a wide range of vertebrate hosts, including mammals, reptiles, birds, and human beings (1–4). Notably, a patient in Russia, *Hepatozoon* gamonts were detected in his blood, presented anemia and icterus (4). In animal hosts, hepatozoonosis can cause moderate to severe clinical symptoms such as anorexia, weight loss, pain, and musculoskeletal disorders, and can be fatal in severe cases (5–7).

To date, over 340 distinct species of *Hepatozoon* have been identified worldwide. Among these, more than 200 species infect large- or middle-size reptiles, and approximately 50 species are known to parasitize mammals (8, 9). Some *Hepatozoon* species exhibit a degree of host specificity (2). For example, *Hepatozoon procyonis* has been detected in the northern raccoon (*Procyon lotor*), *Hepatozoon canis* in the golden jackal (*Canis aureus*), *Hepatozoon felis* in the European wildcat (*Felis silvestris*), *Hepatozoon* cf. *griseisciuri* in the eastern gray squirrel (*Sciurus carolinensis*), and *Hepatozoon erhardovae* in the bank vole (*Clethrionomys glareolus*) (2, 10, 11). In contrast, some *Hepatozoon* species show low host specificity and have been found to infect diverse animal taxa (12). For instance, *Hepatozoon americanum* has been found in the coyote (*Canis latrans*) and hispid cotton rat (*Sigmodon hispidus*), and *Hepatozoon sciuri* has been detected in the European hedgehog (*Erinaceus europaeus*) and red squirrel (*Sciurus vulgaris*) (2, 13).

Xinjiang Uygur Autonomous Region (XUAR), located in northwestern China (73°E-96°E, 34°N-48°N), is the largest provincial-level division (covering 1.66 million km^2^) in the country (14, 15). This region encompasses diverse ecosystems, including deserts, alpine meadows, forests, wetlands, and lacustrine zones (16). Such extensive and varied landscapes provide critical habitats for a wide range of terrestrial wildlife, including 86 rodent species, 49 reptile species, 27 carnivore species, and 14 ruminant species (17–19). Although sporadic molecular detections have been reported, including *Hepatozoon ayorgbor*-like in the great gerbil (*Rhombomys opimus*), *H. canis* in the red fox (*Vulpes vulpes*) and domestic dog (*Canis familiars*), and *H. felis* in the Eurasian lynx (*Lynx lynx*), no systematic survey has been conducted to assess the overall prevalence and genetic diversity of *Hepatozoon* in this region (20–23). Notably, the ecological overlap and predation behavior in this region may facilitate parasite transmission. To assess the zoonotic potential and genetic characteristics of *Hepatozoon* spp. in northwestern China, this study conducted a large-scale molecular investigation. The aim was to explore the prevalence, species distribution and phylogenetic groups of *Hepatozoon* in wildlife and sympatric domestic animals, based on samples collected from 3,037 mammals and 307 reptiles in the study regions.

## 2 Materials and methods

### 2.1 Sample collection and identification

**Figure 1:**
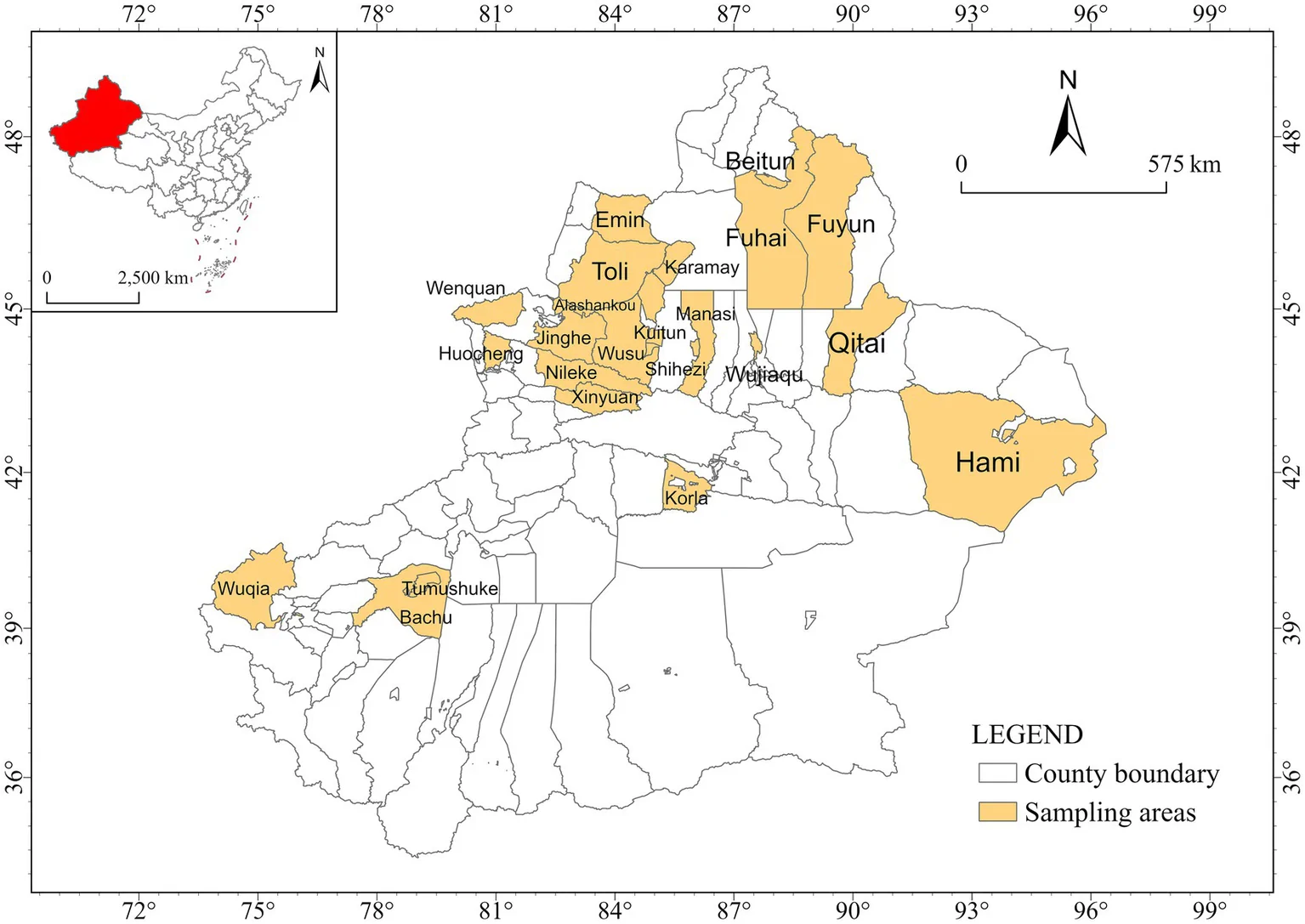
Locations where samples were collected for this study.

From 2018 to 2025, a total of 3,344 samples were collected in XUAR. These comprised 2,304 liver samples sourced from 313 pastured donkeys (imported from Kyrgyzstan) and 1996 wild animals, as well as 1,040 blood samples originating from 838 Kyrgyzstan-imported pastured donkeys and 202 pastured Bactrian camels (Figure 1 and Supplementary Table S1). All animals were euthanized by certified personnel using intravenous injection of sodium pentobarbital at a dose of ≥150 mg/kg, following the national standard of China (GB/T 39760–2021). Subsequently, host species identification was conducted by experienced zoologists, based on both morphological traits and mitochondrial DNA amplification (24–26). Specifically, a 1,242-bp fragment of the cytochrome B (*cytb*) gene was amplified for rodents, and a 650-bp region of cytochrome c oxidase subunit I (*COX1*) for reptiles (Supplementary Table S2) (27, 28). All procedures involving wildlife adhered to the ethical guidelines of the Animal Ethics Committee of Shihezi University. The corresponding sequences were deposited in the GenBank database (*cytb*: OR548119, PQ450151-PQ450153, PQ450155, PQ450156, PQ450158, PQ450159, PQ450162, PQ450163, PQ450165, PQ450167, PQ450168, PQ474772, PQ474773, PQ581939; *COX1*: PQ451734-PQ451736).

### 2.2 DNA extraction

Genomic DNA from each liver sample was individually extracted using the TIANamp Genomic DNA Kit (TIANGEN, Beijing, China) according to the manufacturer’s protocol, while the genomic DNA from each blood sample was extracted using the TIANamp Blood DNA Kit (TIANGEN, Beijing, China). The DNA purity was assessed on a Nanodrop 2000 spectrophotometer (Thermo Fisher Scientific, Waltham, MA, USA). Samples with a DNA concentration of at least 50 ng/μl could be used to further detect *Hepatozoon*.

### 2.3 Molecular detection and sequencing of *Hepatozoon* spp.

*Hepatozoon* species was identified using a polymerase chain reaction (PCR) protocol. This protocol used both forward and reverse primers targeting a 1,100-bp nucleotide fragment of the 18S ribosomal RNA (*18S rRNA*) gene (20). The primers and PCR cycle conditions could be found in Supplementary Table S2. Reagent grade water served as the negative control. DNA extracts of *Hepatozoon* from great gerbils stored in our labs were used as the positive controls (20). The PCR products were purified using the TIANgel Midi Purification Kit (TIANGEN, Beijing, China) and analyzed by Sanger sequencing (Youkang Biotechnology Co., Ltd., Urumqi, China) (16, 29, 30). The sequencing was conducted three times to verify the reproducibility of the results. The nucleotide sequences obtained in this study were edited and then compared against GenBank entries using the BLAST program^1^.

### 2.4 Data analyses

**Figure 2:**
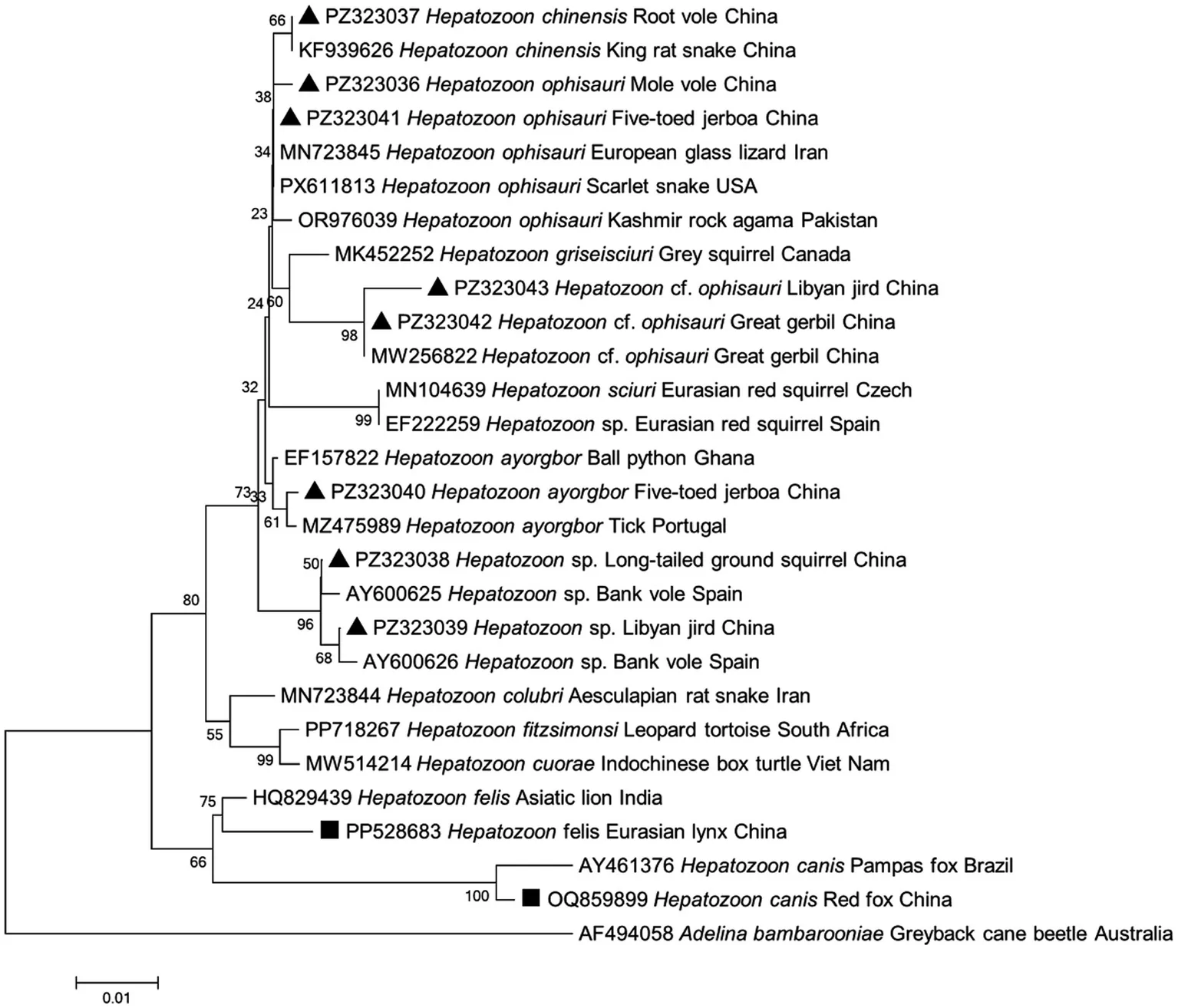
Phylogenetic tree of *Hepatozoon* species was conducted using the neighbor-joining method based on *18S rRNA* gene sequences with 1,000 bootstrap replicates. Novel sequences identified in this study are denoted by black circles and labeled with GenBank accession numbers, host species, and country of origin. Sequences identified in our previous studies are denoted by black squares. *Adelina bambarooniae* was used as an outgroup.

A total of eight *Hepatozoon* sequences from this study were deposited in the GenBank database (*18S rRNA*: PZ323036-PZ323043). Phylogenetic analysis was conducted using MEGA 7.0^2^ software, employing the neighbor-joining method and 1,000 replicates for bootstrap support (Figure 2).

## 3 Results

### 3.1 Host identification

In this study, a total of 3,344 liver or blood samples were collected from 35 animal species across eight orders, including seven mammalian orders (Rodentia, Lagomorpha, Chiroptera, Artiodactyla, Perissodactyla, Carnivora, Primates) and one reptilian order (Squamata) (Supplementary Table S1).

### 3.2 Prevalence of *Hepatozoon* spp.

The overall prevalence of *Hepatozoon* species was 2.5% (84/3344). Infections were detected exclusively in the order Rodentia with a prevalence of 6.5% (84/1301), while no *Hepatozoon* DNA was found in samples from the other six mammalian orders (0/1736) or the reptilian order Squamata (0/307).

### 3.3 Geographic distribution of *Hepatozoon* infections

The geographic distribution of all 84 positive cases is detailed in Supplementary Table S1. Among the positive rodent species, the great gerbil accounted for the majority of infections (68/84, 81.0%), with positive cases detected across four sampling sites (Alashankou, Karamay, Kuitun and Wujiaqu cities). The infection prevalence in the great gerbil varied significantly among sites (*χ*^2^ = 39.68, df = 4, *p* < 0.001), ranging from 0% (0/11) in Qitai County to 56.3% (27/48) in Wujiaqu City.

### 3.4 Species of *Hepatozoon* spp.

Four distinct species were identified in livers of 1,301 rodents, including *Hepatozoon chinensis* (*n* = 1), *Hepatozoon ayorgbor* (*n* = 7), *Hepatozoon ophisauri* (*n* = 3) and *Hepatozoon* cf. *ophisauri* (*n* = 70). In addition, an unnamed *Hepatozoon* sp. (*n* = 3) that could not be assigned to any known species was detected (shown in Table 1 and Supplementary Table S1).

**Table 1:** *Hepatozoon* species detected in this study.

Host species	Gene	*Hepatozoon* species/genotype detected	GenBank number	Similar sequences percent identity and GenBank number	Source reference of the similar sequence
*Ellobius tancrei*	*18S rRNA*	*Hepatozoon ophisauri*	PZ323036	*Hepatozoon ophisauri* 903/904 (99.89%) MN723845	(38)
*Microtus oeconomus*	*18S rRNA*	*Hepatozoon chinensis*	PZ323037	*Hepatozoon chinensis* 867/878 (98.75%) KF939626	(34)
*Spermophilus undulatus*	*18S rRNA*	*Hepatozoon* sp.	PZ323038	*Hepatozoon* sp. 834/840 (99.29%) AY600625	(40)
*Myodes rutilus*	*18S rRNA*	*Hepatozoon* sp.	PZ323039	*Hepatozoon* sp. 919/930 (98.82%) AY600626	(40)
*Allactaga sibirica*	*18S rRNA*	*Hepatozoon ayorgbor*	PZ323040	*Hepatozoon ayorgbor* 986/997 (98.90%) EF157822	(36)
*Allactaga sibirica*	*18S rRNA*	*Hepatozoon ophisauri*	PZ323041	*Hepatozoon ophisauri* 987/989 (99.80%) MZ723845	(38)
*Rhombomys opimus*	*18S rRNA*	*Hepatozoon* cf. *ophisauri*	PZ323042	*Hepatozoon* cf. *ophisauri* 969/972 (99.69%) MW256822	/
*Meriones libycus*	*18S rRNA*	*Hepatozoon* cf. *ophisauri*	PZ323043	*Hepatozoon* cf. *ophisauri* 961/969 (99.17%) MW256822	/

Molecular analysis in this study indicated that (i) *Hepatozoon chinensis* detected from the root vole (*Microtus oeconomus*) showed 98.8% identity to *H. chinensis* reported in China from king rat snakes (*Elaphe carinata*) (GenBank accession number: KF939626); (ii) *Hepatozoon ayorgbor* detected from the five-toed jerboa (*Allactaga sibirica*) had 98.9% (986/997) identity to an isolate of *H*. *ayorgbor* from ball pythons (*Python regius*) sampled in Ghana (EF157822); (iii) *Hepatozoon ophisauri* detected from the mole vole (*Ellobius talpinus*) and the five-toed jerboa exhibited 99.8% (987/989) and 99.9% (903/904) sequence identities to an isolate of *H*. *ophisauri* from European glass lizards (*Pseudopus apodus*) sampled in Iran (MN723845); (iv) *Hepatozoon* cf. *ophisauri* detected from the great gerbil and the Libyan jird (*Meriones libycus*) showed 99.2% (961/969) and 99.7% (969/972) identities to *H*. cf. *ophisauri* reported from the great gerbil in China (MW256822); and (v) the unnamed *Hepatozoon* sp. detected from the long-tailed ground squirrel (*Spermophilus undulatus*) and the red-backed vole (*Myodes rutilus*) exhibited 99.3% (834/840) and 98.8% (919/930) sequence identities to *Hepatozoon* sp. reported from bank voles in Spain, respectively (AY600625 and AY600626).

The above species of *Hepatozoon* were confirmed by phylogenetic analysis of the *18S rRNA* gene. The phylogenetic tree constructed using the *18S rRNA* gene is shown in Figure 2. To further validate the species identification, we performed multiple sequence alignments using DNAMAN (Supplementary Figure S1).

## 4 Discussion

*Hepatozoon* was detected in 3,344 liver or blood specimens, belonging to 25 mammal and 10 reptile species in northwestern of China. Notably, all positive samples were detected exclusively in Rodentia with a prevalence of 6.5% (84/1301), while no *Hepatozoon* DNA was found in the other six mammalian orders (Lagomorpha, Chiroptera, Artiodactyla, Perissodactyla, Carnivora, and Primates) and the reptilian order Squamata. With exception of our previous report on *H. canis* in the red fox and *H. felis* in the Eurasian lynx, five additional *Hepatozoon* species (*H. chinensis*, *H*. *ayorgbor*, *H*. *ophisauri*, *H*. cf. *ophisauri* and an unnamed *Hepatozoon* sp.) were detected in this study (21, 23). Here we first recorded *Hepatozoon* infection in several rodent species, such as *H. chinensis* in the root vole, *H*. *ayorgbor* in the five-toed jerboa, *H*. *ophisauri* in the mole vole and five-toed jerboa, *H*. cf. *ophisauri* in the great gerbil and Libyan jird, and *Hepatozoon* sp. in the long-tailed ground squirrel. Among the five *Hepatozoon* genetic variants identified in this study, we observed two distinct ecological patterns: three species (*H. chinensis*, *H*. *ayorgbor* and *H*. *ophisauri*) originally associated with reptiles were detected for the first time in rodents (representing host range expansion), while two other species (*H*. cf. *ophisauri* and *Hepatozoon* sp.) have previously been documented in rodents and show varying degrees of host specificity.

Rodents serve as critical intermediate or paratenic hosts in the epidemiology of *Hepatozoon* species (31). Those *Hepatozoon* taxa are associated with rodents and reptiles cluster within clade I of the phylogenetic tree, and they have co-evolved with their vertebrate hosts, with spillover infections occurring among species that share phylogenetic relationships (32, 33). In this study, we detected five *Hepatozoon* species across seven rodent species from three families, which indicates that *Hepatozoon* parasites are prevalent across a broad range of host lineages in northwestern China. More importantly, our results showed that the same *Hepatozoon* species were found in different rodent species co-existing in the same geographical habitat, as exemplified by the detection of *Hepatozoon* cf. *ophisauri* in both the great gerbil and Libyan jird. This finding confirms the potential for cross-species transmission of *Hepatozoon* among sympatric hosts within a shared environment. The diverse desert, semi-desert, and montane ecosystems of XUAR offer highly overlapping habitats for multiple rodent and reptile species, and such niche overlap may further facilitate pathogen transmission among sympatric hosts (16–19).

*Hepatozoon chinensis* was first described in the king rat snake in China (34). In the present study, this species was identified in a root vole, with 98.8% sequence identity to the previously reported isolate (KF939626). This finding represents the first record of *H. chinensis* in a mammalian host, thereby expanding the known host range from reptiles to mammals and strongly suggesting that wild rodents can function as parasitic or intermediate hosts in the transmission cycle. Further studies are needed to elucidate the specific ecological and vector-mediated pathways involved.

*Hepatozoon ayorgbor* was originally described from ball pythons in Africa (35). Experimental studies have demonstrated that rodents can serve as intermediate or paratenic hosts: ball pythons became infected after consuming mice experimentally inoculated with the parasite (36). In Europe, *H*. *ayorgbor* has also been detected in wild rodents, including yellow-necked mice (*Apodemus flavicollis*) and wood mice (*Apodemus sylvaticus*) in Croatia (7). In present study, this species was identified for the first time in five-toed jerboas, with 98.9% sequence identity to the Ghana isolate (EF157822) from the ball python. Together with previous experimental and field evidence, the detection of *H*. *ayorgbor* in the five-toed jerboa lends further support to the involvement of rodents in the natural history of this parasite. Specifically, this finding indicates that the known rodent host range of *H*. *ayorgbor* may extend to the five-toed jerboa in Central Asia, complementing earlier records from *Apodemus* species in Europe.

*Hepatozoon ophisauri*, a snake-associated species, was first reported in European glass lizards in Armenia, and subsequently detected in glass lizards in Iran, and in Tanezumi rats (*Rattus tanezumi*) in Malaysia (37–39). In present study, *H*. *ophisauri* was detected for the first time in a mole vole and two five-toed jerboas, with 99.8–99.9% identity with the Iranian isolate (MN723845). This finding further supports that reptile-associated *Hepatozoon* species can utilize taxonomically diverse rodents as intermediate or paratenic hosts, potentially facilitating cross-Order transmission when ecological opportunities arise.

*Hepatozoon* cf. *ophisauri* has been previously identified in the great gerbil in China (GenBank accession number: MW256822). In the present study, sequences of this species showed 99.2–99.7% identity to the previously reported isolate (MW256822). In addition, phylogenetic analysis confirmed that all *H*. cf. *ophisauri* sequences from this study clustered with MW256822 in a well-supported clade (Figure 2). The high prevalence and apparent host specificity of *H*. cf. *ophisauri* in Gerbillinae rodents suggest that this variant may have established a stable association with these hosts, potentially involving specific vector species adapted to desert and semi-desert ecosystems.

In addition to the four known species, an unnamed *Hepatozoon* sp. was detected in long-tailed ground squirrels and a red-backed vole. Their *18S rRNA* sequences exhibited 99.3% (834/840) and 98.8% (919/930) identity, respectively, to *Hepatozoon* sp. isolates reported from bank voles in Spain (AY600625 and AY600626) (40). Phylogenetically, these sequences clustered together in a well-supported clade with AY600625 and AY600626 (Figure 2). Although these Spanish isolates have been suggested to be closely related to *H*. *erhardovae*, a common parasite of bank voles in Europe, their exact taxonomic status remains under debate, with recent evidence indicating potential species-level differences between the two variants (30, 41). Notably, the positive samples originated from two rodent families with distinct ecological niches: long-tailed ground squirrels (Sciuridae) inhabit alpine meadows, while red-backed voles (Cricetidae) are predominantly found in boreal forests (42, 43). This result suggests that this unnamed *Hepatozoon* sp. may have the capacity to infect multiple rodent species across different ecological habitats.

This study has several limitations. First, the small sample sizes for several families (e.g., Bovidae *n* = 1, Cercopithecidae *n* = 1, Suidae *n* = 5, and Mustelidae *n* = 7) involed in this study, which led to no *Hepatozoon* detected in these samples. Second, only five *Hepatozoon* species were screenouted in rodents, as for only seven rodent species were detected. Third, systematic risk factor analyses (e.g., age, sex, season, habitat type) were not performed. In the future, we should pay more attention to these limitations, and obtain more comprehensive knowledge to *Hepatozoon* infection especially in wildlife.

## 5 Conclusion

In this study, a large-scale investigation of *Hepatozoon* infections was conducted by screening 3,344 samples from 35 animal species across XUAR, northwestern China. Overall, five *Hepatozoon* species were identified, including four known species (*H. chinensis*, *H*. *ayorgbor*, *H*. *ophisauri*, *H*. cf. *ophisauri*) and one unnamed *Hepatozoon* sp. All positive samples were exclusively detected in rodents, which represents new host records: *Hepatozoon chinensis* in the root vole, *H*. *ayorgbor* in the five-toed jerboa, *H*. *ophisauri* in the mole vole and five-toed jerboa, *H*. cf. *ophisauri* in the great gerbil and Libyan jird, and *Hepatozoon* sp. in the long-tailed ground squirrel. These findings expand the known host range of *Hepatozoon* species and their geographic distribution.

## Data Availability

The datasets presented in this study can be found in online repositories. The names of the repository/repositories and accession number(s) can be found at: https://www.ncbi.nlm.nih.gov/, Rodent species *cytb*: OR548119, PQ450151-PQ450153, PQ450155, PQ450156, PQ450158, PQ450159, PQ450162, PQ450163, PQ450165, PQ450167, PQ450168, PQ474772, PQ474773, PQ581939; reptile species *COX1*: PQ451734-PQ451736; *Hepatozoon 18S rRNA*: PZ323036-PZ323043.

## Footnotes

1
http://www.ncbi.nlm.nih.gov/blast/

2
http://www.megasoftware.net

## Supplementary material

SUPPLEMENTARY FIGURE 1 Multiple sequence alignment of the partial *18S rRNA* gene fragments of the *Hepatozoon* species identified in this study [*Hepatozoon chinensis* **(A)**, *Hepatozoon ayorgbor* **(B)**, *Hepatozoon ophisauri* **(C)**, *Hepatozoon* cf. *ophisauri* **(D)**, and *Hepatozoon* sp. **(E)**].

SUPPLEMENTARY TABLE S1 The sources of the samples and the detection of Hepatozoon species in this study.

SUPPLEMENTARY TABLE S2 The data of **(A)** primers and **(B)** cycling conditions used in this study. The genetic markers that served for identification of species were as follows: cytb for rodent species, COX1 for reptile species, and 18S rRNA for Hepatozoon spp.
