# Supplementary material for: Molecular detection and phylogeny of Hepatozoon species in a wide spectrum of hosts from northwestern China
Source: Front Vet Sci. 2026 Aug 31;13:1897152. doi: 10.3389/fvets.2026.1897152 (PMC13572295; doi:10.3389/fvets.2026.1897152)
Supplement: Supplementary file 1 [file Table_1.docx]

**Additional file1: Table S1.** The source of the samples and the detection of *Hepatozoon* species in this study.

| **Animal** | **Sample type** | **No. examined** | **No. Positive**  **(%)** | **Species (No. of positive samples)** | **GenBank number** | **Sampling site**  **(No. of samples)** | **Positive sampling sites (No. of positive samples)** | **Collection time**  **(year)** | **Permit** |
| --- | --- | --- | --- | --- | --- | --- | --- | --- | --- |
| Mammalian | | | | | | | | | |
| Rodentia | | | | | | | | | |
| *Rhombomys opimus* | liver | 262 | 68  (26.0) | *Hepatozoon* cf. *ophisauri*  (n=68) | PZ323042 | Alashankou City  (n=86)  Huocheng county  (n=1)  Karamay City  (n=78)  Kuitun City  (n=38)  Qitai County  (n=11)  Wujiaqu City  (n=48) | Alashankou City  (n=19)  Karamay City  (n=8)  Kuitun City  (n=14)  Wujiaqu City  (n=27) | 2022-2024 | Approval No. A2022-029-01 |
| *Meriones*  *libycus* | liver | 145 | 2  (1.4) | *Hepatozoon* cf. *ophisauri*  (n=2) | PZ323043 | Alashankou City  (n=50)  Kuitun City  (n=93)  Manasi County  (n=2) | Kuitun City  (n=2) | 2018, 2023 | Approval Nos. AECSU2015-01 and A2022-029-01 |
| *Meriones tamariscinus* | liver | 5 | 0 | / | / | Huocheng County  (n=1)  Kuitun City  (n=1)  Manasi County  (n=3) | / | 2023, 2024 | Approval No. A2022-029-01 |
| *Meriones meridianus* | liver | 10 | 0 | / | / | Manasi County  (n=8)  Qitai County  (n=1)  Bachu County  (n=1) | / | 2021, 2022 | Approval Nos. A2018-143-01 and A2022-029-01 |
| *Spermophilus undulatus* | liver | 573 | 2  (0.9) | *Hepatozoon* sp.  （n=2） | PZ323038 | Fuhai County  (n=185)  Fuyun County  (n=188)  Jinghe County  (n=149)  Wenquan County  (n=51) | Fuhai County  (n=2) | 2020, 2023, 2024 | Approval Nos. A2018-143-01 and A2022-029-01 |
| *Spermophilus erythrogenys* | liver | 253 | 0 | / | / | Emin County  (n=37)  Karamay City  (n=188)  Toli County  (n=28) | / | 2022, 2023 | Approval No. A2022-029-01 |
| *Marmota himalayana* | liver | 1 | 0 | / | / | Fuhai County  (n=1) | / | 2020 | Approval No. A2018-143-01 |
| *Tamias*  *Sibiricus* | liver | 1 | 0 | / | / | Fuhai County  (n=1) | / | 2020 | Approval No. A2018-143-01 |
| *Microtus oeconomus* | liver | 1 | 1  (100.0) | *Hepatozoon chinensis*  (n=1) | PZ323037 | Fuhai County  (n=1) | Fuhai County  (n=1) | 2019 | Approval No. A2018-143-01 |
| *Ellobius talpinus* | liver | 11 | 1  (9.1) | *Hepatozoon ophisauri*  (n=1) | PZ323036 | Fuhai County  (n=11) | Fuhai County  (n=1) | 2019 | Approval No. A2018-143-01 |
| *Ondatra*  *zibethicus* | liver | 1 | 0 | / | / | Korla City  (n=1) | / | 2025 | Approval No. A2022-029-01 |
| *Allactaga*  *sibirica* | liver | 27 | 9  (33.3) | *Hepatozoon ayorgbor*  (n=7)  *Hepatozoon ophisauri*  (n=2) | PZ323040, PZ323041 | Qitai County  (n=27) | Qitai County  (n=9) | 2025 | Approval No. A2022-029-01 |
| *Dipus sagitta* | liver | 1 | 0 | / | / | Manasi County  (n=1) | / | 2023 | Approval No. A2022-029-01 |
| *Mus*  *musculus* | liver | 8 | 0 | / | / | Fuhai County  (n=8) | / | 2023 | Approval No. A2022-029-01 |
| *Myodes rutilus* | liver | 2 | 1  (50.0) | *Hepatozoon* sp.  （n=1） | PZ323039 | Fuhai County  (n=2) | Fuhai County  (n=1) | 2019 | Approval No. A2018-143-01 |
| Lagomorpha | | | | | | | | | |
| *Ochotona pallasi* | liver | 92 | 0 | / | / | Qitai County  (n=92) | / | 2022, 2023 | Approval No. A2022-029-01 |
| Chiroptera | | | | | | | | | |
| *Pipistrellus pipistrellus* | liver | 267 | 0 | / | / | Xinyuan County  (n=267) | / | 2020, 2021 | Approval No. A2018-143-01 |
| Artiodactyla | | | | | | | | | |
| *Sus*  *scrofa* | liver | 5 | 0 | / | / | Bachu County  (n=4)  Nileke County  (n=1) | / | 2019, 2024 | Approval No. A2018-143-01  Approval No. A2022-029-01 |
| *Procapra*  *przewalskii* | liver | 1 | 0 | / | / | Wujiaqu City  (n=1) | / | 2024 | Approval No. A2022-029-01 |
| *Camelus*  *bactrianus* | blood | 202 | 0 | / | / | Qitai County  (n=202) | / | 2025 | Approval No. A2022-029-01 |
| Perissodactyla | | | | | | | | | |
| *Equus*  *asinus* | liver  blood | 1151 | 0 | / | / | Kyrgyz Republic  (n=838)  Wuqia County  (n=313) | / | 2023, 2024 | Approval No. A2022-029-01 |
| Carnivora | | | | | | | | | |
| *Vormela*  *peregusna* | liver | 6 | 0 | / | / | Karamay City  (n=1)  Manasi County  (n=2)  Qitai County  (n=3) | / | 2020-2024 | Approval Nos. A2018-143-01 and A2022-029-01 |
| *Canis familiaris* | liver | 10 | 0 | / | / | Korla City  (n=10) | / | 2025 | Approval No. A2022-029-01 |
| *Meles*  *meles* | liver | 1 | 0 | / | / | Wujiaqu City  (n=1) | / | 2024 | Approval No. A2022-029-01 |
| Primates | | | | | | | | | |
| *Cercopithecidae* | liver | 1 | 0 | / | / | Shihezi City  (n=1) | / | 2024 | Approval No. A2022-029-01 |
| Reptilian | | | | | | | | | |
| Squamata | | | | | | | | | |
| *Eremias velox* | liver | 54 | 0 | / | / | Huocheng County  (n=15)  Jinghe County  (n=2)  Karamay City  (n=9)  Shihezi City  (n=1)  Tumushuke City  (n=8)  Wusu City  (n=19) | / | 2023, 2024 | Approval No. A2022-029-01 |
| *Eremias vermiculata* | liver | 7 | 0 | / | / | Huocheng County  (n=2)  Karamay City  (n=2)  Wusu City  (n=3) | / | 2023 | Approval No. A2022-029-01 |
| *Trapelus sanguinolentus* | liver | 6 | 0 | / | / | Huocheng County  (n=6) | / | 2023 | Approval No. A2022-029-01 |
| *Phrynocephalus axillaris* | liver | 80 | 0 | / | / | Hami City  (n=31)  Tumushuke City  (n=49) | / | 2023 | Approval No. A2022-029-01 |
| *Phrynocephalus forsythii* | liver | 22 | 0 | / | / | Tumushuke City  (n=22) | / | 2023 | Approval No. A2022-029-01 |
| *Phrynocephalus grumgrizimailoi* | liver | 100 | 0 | / | / | Beitun City  (n=1)  Huocheng County  (n=33)  Karamay City  (n=53)  Manasi County  (n=4)  Qitai County  (n=5)  Shihezi City  (n=2)  Wusu City  (n=2) | / | 2023 | Approval No. A2022-029-01 |
| *Phrynocephalus guttatus* | liver | 30 | 0 | / | / | Jinghe County  (n=7)  Manasi County  (n=13)  Wusu City  (n=10) | / | 2023, 2024 | Approval No. A2022-029-01 |
| *Phrynocephalus versicolor* | liver | 1 | 0 | / | / | Qitai County  (n=1) | / | 2023 | Approval No. A2022-029-01 |
| *Phrynocephalus helioscopus* | liver | 1 | 0 | / | / | Qitai County  (n=1) | / | 2023 | Approval No. A2022-029-01 |
| *Laudakia stoliczkana* | liver | 6 | 0 | / | / | Qitai County  (n=6) | / | 2023 | Approval No. A2022-029-01 |
| Total | liver  blood | 3344 | 84  (2.5) | *Hepatozoon* cf. *ophisauri*  (n=70)  *Hepatozoon* sp.  (n=3)  *Hepatozoon chinensis*  (n=1)  *Hepatozoon ophisauri*  (n=3)  *Hepatozoon ayorgbor*  (n=7) | PZ323036-PZ323043 | / | / | 2018-2025 | Approval Nos. AECSU2015-01, Approval No. A2018-143-01 and Approval No. A2022-029-01 |
