# Supplementary material for: Molecular detection and phylogeny of Hepatozoon species in a wide spectrum of hosts from northwestern China
Source: Front Vet Sci. 2026 Aug 31;13:1897152. doi: 10.3389/fvets.2026.1897152 (PMC13572295; doi:10.3389/fvets.2026.1897152)
Supplement: Supplementary file 2 [file Table_2.docx]

**Additional file 2: Table S2.** The data of (A) primers and (B) cycling conditions used in this study. The genetic markers that served for identification of species were as follows: *cytb* for rodent species, *COX1* for reptile species and *18S rRNA* for *Hepatozoon* spp.

**Table S2A.** The data of primers used in this study.

| **Target taxon** | **Target gene** | **Primer sequence (5’-3’)** | **Fragment size (bp)** | **Reference** |
| --- | --- | --- | --- | --- |
| Rodents | *cytb* | F: ACCAATGACATGAAAAATCATCGTT  R: TCTCCATTTCTGGTTTACAAGAC | 1242 bp | [27] |
| Lizards | *COX1* | F: TYTCWACWAAYCAYAAAGAYATCGG  R: ACYTCRGGRTGRCCRAARAATCA | 650 bp | [28] |
| *Hepatozoon* | *18S rRNA* | F: ATGGCTCATTACAACAGT  R: AAGTTTCAGCCTTGCGACCATACTC | 1100 bp | [20] |

**Table S2B.** The data of cycling conditions used in this study.

| **Gene** | **Initial denaturation (℃, sec)** | **Denaturation (℃, sec)** | **Annealing (℃, sec)** | **Extension (℃, sec)** | **Cycle number** | **Final extension (℃, sec)** |
| --- | --- | --- | --- | --- | --- | --- |
| *cytb* | 94, 300 | 94, 30 | 55, 60 | 72, 60 | 30 | 72, 600 |
| *COX*1 | 94, 180 | 94, 30 | 50, 30 | 72, 30 | 40 | 72, 600 |
| *18S rRNA* | 95, 300 | 95, 40 | 58, 40 | 72, 60 | 40 | 72, 480 |
